# 1501. Characterizing Engagement in Care and STI Screenings among DC Cohort Participants with HIV and Mpox

**DOI:** 10.1093/ofid/ofad500.1336

**Published:** 2023-11-27

**Authors:** Lauren O’Connor, Morgan Byrne, Elisabeth Anderson, Debra A Benator, Jose Lucar, Michael A Horberg, Rachel V Denyer, Rachel Lee, Amanda Derryck Castel, Anne K Monroe

**Affiliations:** George Washington University Milken Institute School of Public Health, Washington, District of Columbia; The George Washington University, Washington, DC; George Washington University Milken Institute School of Public Health, Washington, District of Columbia; Washington DC VA Medical Center, Washington DC, DC; The George Washington University, Washington, DC; Kaiser Permanente Mid-Atlantic States Mid-Atlantic Permanente Medical Group, Rockville, Maryland; Washington DC VA Medical Center/ George Washington University, Washington, DC; The George Washington University Hospital, Washington, District of Columbia; The George Washington University Milken School of Public Health, Washington, DC; The George Washington University, Washington, DC

## Abstract

**Background:**

A high proportion of people with HIV (PWH) in the 2022-2023 mpox outbreak (approx. 40% of US cases) has raised questions about the association between HIV and mpox. Increased encounters with the healthcare system may be contributing to this association. This study sought to elucidate whether detection bias drives the relationship between mpox and HIV by determining whether engagement in care and STI screening are associated with having an mpox diagnosis.

**Methods:**

We conducted a 5:1 matched case-control study among DC Cohort participants, a longitudinal cohort of PWH receiving care in Washington, DC. Cases were matched on age (+/-5 years), gender, race/ethnicity, HIV transmission risk factor, time since HIV diagnosis (+/-5 years), years enrolled (+/-3 years), and HIV care site (community or hospital) with up to 5 controls. Engagement in care was defined as two or more CD4 or HIV RNA tests at least 90 days apart during the last year. STI screening was any test for gonorrhea, chlamydia, or syphilis in the past year. Cases were participants with an mpox diagnosis identified by providers or an ICD10 code in the EHR. We used unadjusted conditional logistic regression to assess the relationship between engagement in care, STI screening, and mpox diagnosis.

**Results:**

We identified 68 cases of mpox in the DC Cohort and 65 could be matched to 300 controls (n = 365). Participants were majority male (98.9%), Black (70.7%), MSM (89.3%), and 51.5% of participants had an STI diagnosis since cohort enrollment. Cases were significantly more likely to be engaged in care (50.8% vs. 26.3%; **OR: 3.24**), receive an STI screening (69.2% vs. 41.3%; **OR: 3.45**), have a historical STI (72.3% vs. 47.0%; **OR: 3.58**), and have a CD4 count < 500 (40.4% vs. 21.9%; **OR: 2.51**).

**Figure:**
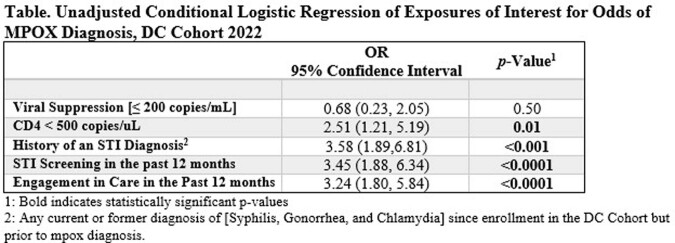

**Conclusion:**

Among a cohort of PWH, those with an mpox diagnosis were significantly more likely to be engaged in care and received an STI screening in the year prior, suggesting increased encounters for HIV care may account for a higher rate of PWH among mpox cases. The high proportion of CD4 < 500 among mpox cases suggests more clinically evident manifestations in this population. Future analyses should examine unmeasured behavioral factors to explain the association between mpox diagnosis, increased engagement in care and STI screening.

**Disclosures:**

**All Authors**: No reported disclosures

